# An exploratory study of behavioral, cognitive, physiological, and microbiota profiles in senior dogs

**DOI:** 10.3389/fnbeh.2026.1689807

**Published:** 2026-02-24

**Authors:** Begum Saral, Durmus Atilgan, Deniz Adiay, Nazlican Filazi, Hakan Ozturk, Gorkem Kismali, Goncalo Da Graca Pereira, Aykut Ozkul, Yasemin Salgirli Demirbas

**Affiliations:** 1 Department of Physiology, Faculty of Veterinary Medicine, Ankara University, Ankara, Türkiye; 2 Institute of Health Sciences, Ankara University, Ankara, Türkiye; 3 Department of Internal Medicine, Faculty of Veterinary Medicine, Ankara University, Ankara, Türkiye; 4 Department of Virology, Faculty of Veterinary Medicine, Hatay Mustafa Kemal University, Antakya, Hatay, Türkiye; 5 Department of Virology, Faculty of Veterinary Medicine, Ankara University, Ankara, Türkiye; 6 Department of Biochemistry, Faculty of Veterinary Medicine, Ankara University, Ankara, Türkiye; 7 Egas Moniz Center for Interdisciplinary Research (CiiEM), Egas Moniz School of Health and Science, Caparica, Almada, Portugal; 8 Department of Psychology, Faculty of Arts, University of Prince Edward Island, Charlottetown, PE, Canada

**Keywords:** brain derived neurotrophic factor, canine cognitive decline, C-BARQ, chronic pain, microbiota-gut-brain axis

## Abstract

**Introduction:**

Aging in dogs is a multifactorial process involving behavioral, cognitive, immunological, and microbiota-related changes, yet distinguishing healthy from pathological aging remains challenging. This exploratory study aimed to evaluate physiological indicators of health by integrating pain evaluation and cognitive testing in senior companion dogs.

**Methods:**

Eighteen companion dogs aged ≥8 years underwent standardized behavioral and cognitive evaluations (Mini C-BARQ, DISHAA, object choice test), chronic pain assessment (Helsinki Chronic Pain Index), and quality-of-life (QoL) scoring. Hematological parameters, serum brain-derived neurotrophic factor (BDNF), and Th1/Th2 ratios were measured as physiological indicators, while fecal samples were analyzed via 16S rRNA sequencing for microbiota profiling.

**Results:**

All dogs scored above the chronic pain threshold (mean HCPI: 28.72), although caregiver-reported QoL ratings suggested good overall wellbeing. Cognitive testing yielded low average scores on the DISHAA (mean: 9.05), with only one dog showing mild cognitive decline; however, mean performance on the object choice test was low (1.94/5). Mean serum BDNF concentration was 0.154 ng/dL (SD: 0.082) and correlated positively with red blood cell (RBC) count and negatively with MCV, MCH, and MCHC (*p* ≤ 0.05). Immune profiling patterns suggested Th2 polarization. The gut microbiota was dominated by Firmicutes and Bacteroidetes. Principal Component Analysis (PCA) identified two primary dimensions of biological variation: a pain–immune–microbiota axis, defined by higher chronic pain scores, Th2 polarization, increased Prevotella abundance, and higher DISHAA scores, and a second component reflecting microbiota compositional variation.

**Discussion:**

These preliminary findings highlight potential interactions between pain, microbiota composition, and immune dysregulation, suggesting their possible utility as candidate indicators for differentiating healthy from pathological aging in dogs.

## Introduction

Aging is a complex, natural and ontogenetic process characterized by a progressive decline in an organism’s ability to maintain homeostasis in response to internal and external stressors (McKenzie et al., 2022). Aging affects multiple biological systems and is often accompanied by both physiological and behavioral changes. In companion animals, while “geriatric” typically refers to the final 25% of their breed-specific life expectancy (Rajapaksha, 2018), “senior” encompasses the broader period of age-related changes which capture early-to-moderate aging processes (Harvey et al., 2021).

Age-related behavioral changes exist on a continuum and there remains considerable debate surrounding the distinction between normal, i.e., physiological/healthy aging and pathological aging (Gladyshev and Gladyshev, 2016). In dogs, various behavioral assessments including those evaluating attention (Wallis et al., 2014), problem-solving (Kubinyi and Iotchev, 2020) and reversal learning (Mongillo et al., 2013) have revealed performance declines in senior dogs when compared to younger individuals, even in the absence of overt cognitive dysfunction. However, differentiating between normal cognitive aging and early stages of cognitive dysfunction syndrome (CDS) is currently based solely on behavioral assessments, which remains challenging (Salvin et al., 2011; Landsberg and Malamed, 2017). Thus, identifying physiological markers that parallel behavioral changes is necessary for differentiating healthy and pathological aging in dogs.

In senior dogs, immunological changes parallel to many of the age-related neurobiological alterations. It was previously reported that both Th1 and Th2 lymphocyte subsets increase with age in dogs, but the rise in Th1 is more pronounced (Horiuchi et al., 2007). Similarly, elderly humans often show increased Th1 percentages compared to younger individuals (Sakata-Kaneko et al., 2000). Those alterations may contribute to immunosenescence, the age-related decline in immune function (Liu et al., 2023). Chronic low-grade inflammation, or “inflammaging” is further commonly associated with aging and may contribute to Th1-driven inflammation (Martynova et al., 2022). This process is strongly associated with age-related conditions such as Alzheimer’s disease and cardiovascular disorders (Xia et al., 2016; Giunta et al., 2008).

The gut microbiota plays an essential role in maintaining both immune and brain health, with increasing attention on its impact on brain health and cognition through the gut-brain axis (Honda and Littman, 2016). Human literature suggests gut microbiota as an important indicator of healthy aging, as dysbiosis, i.e., a shift in the composition of microbiota, is associated with an increased risk of various diseases due to both local and systemic inflammation that occurs with aging (Ragonnaud and Biragyn, 2021; Gupta et al., 2024). Dogs show greater microbial similarity to humans in both composition and functional capacity than traditional animal models such as mice or pigs (Deng and Swanson, 2014; Coelho et al., 2018). Studies have further demonstrated that cohabiting dogs and humans can even share similar microbial profiles (Misic et al., 2015). Senior companion dogs may therefore represent a relevant translational model for studying aging-related gut–brain interactions, given their shared environmental exposures and gut microbiota characteristics with humans. In dogs, aging is accompanied by specific microbial changes including reduced diversity and increased levels of lactobacilli (Mizukami et al., 2019). A recent study also reported age-related shifts in gut microbiota composition in senior dogs, which supports the idea that the gut microbiota changes with age (Fernández-Pinteño et al., 2023). However, there remains a lack of data on how these changes can be effectively used to define or predict healthy aging in dogs.

Pain is another critical factor when assessing behavioral and quality-of-life changes in aging dogs. As pain is closely associated with age and the aging process (Gagliese, 2009), it can be recognized as a key indicator of healthy aging. Both acute and chronic pain can complicate the behavioral profiles of senior dogs by altering activity patterns and play behavior, increasing irritability and anxiety, and even precipitating sudden-onset aggression (Camps et al., 2012; Mills et al., 2020; Malkani et al., 2024). Because pain-related behaviors are often misinterpreted as normal signs of aging, these changes should be evaluated alongside reliable quality-of-life assessments and, whenever possible, in conjunction with physiological parameters (Belshaw and Yeates, 2018; Pais et al., 2024). For instance, studies indicate that BDNF levels are generally lower in individuals experiencing chronic pain compared to healthy controls, and such reductions may be linked to immune cell alterations as well as psychological factors such as anxiety and depression (Dimmek et al., 2021; Ortolá et al., 2025).

Brain-derived neurotrophic factor (BDNF), a member of the neurotrophin family, plays a key role in regulating neuronal survival, differentiation and synaptic plasticity (Huang and Reichardt, 2001). In human studies, reductions in BDNF, mRNA and protein levels have been observed even during mild cognitive impairment and found to be correlated with cognitive decline (Peng et al., 2005). Both human and animal studies have linked reduced BDNF levels with synaptic loss and cognitive deterioration associated with aging and neurodegeneration (Peng et al., 2005; Keifer, 2022). BDNF has indeed been considered a potential treatment option for neurodegenerative diseases accompanied by cognitive decline, such as Alzheimer’s disease (Lu et al., 2013; Gliwińska et al., 2023).

Given the multifaceted nature of aging, a comprehensive assessment that integrates behavioral observations, cognitive testing, pain evaluation and physiological markers is essential for identifying indicators of healthy aging. This exploratory study aims to comprehensively evaluate age-related changes in senior dogs by measuring behavioral and cognitive function, pain and quality of life status, serum BDNF levels, Th1/Th2 cytokine concentrations, and gut microbial profiles.

Based on this literature, we formulated the following hypotheses: (1) senior dogs would exhibit altered BDNF levels; (2) pain would be prevalent; (3) immune profiles would show altered Th1/Th2 balance; and (4) gut microbiota composition would display age-associated changes. This exploratory study integrates behavioral, cognitive, immunological, and microbiota assessments to identify candidate markers for distinguishing healthy from pathological aging in dogs.

## Materials and methods

This study was conducted with the approval of the Ankara University Animal Experiments Local Ethics Committee (Approval number: 2022-22-133).

### Animals

A total of 20 senior dogs (aged ≥8 years; all neutered) that visited a private veterinary clinic in Ankara for routine health checks were initially included in the study (Table 1). Two of the dogs were later excluded due to health issues. All dogs were client-owned, living in home environments with regular human contact and had no diagnosed acute or chronic systemic diseases at the time of enrollment based on caregiver reports and routine veterinary examination. While dogs were considered healthy at study entry, subclinical conditions such as undiagnosed osteoarthritis may have been present, as subsequently indicated by pain assessments. Accordingly, the term “healthy” is used to describe the absence of clinically diagnosed disease rather than the confirmed absence of underlying pathology. All dogs were fed commercial diets; however, diet brands, formulations, and feeding regimens (once or twice daily) varied across individuals.

**Table 1:** Demographics of the dogs.

No	Breed	Gender	Age (years)
1	Labrador retriever	Female	9
2	Labrador retriever	Male	9
3	German shepherd	Female	14
4	Golden retriever	Female	10
5	Terrier mix (unknown)	Male	8
6	Mixed breed (mongrel)	Female	14
7	Beagle	Female	13
8	Rottweiler	Male	10
9	Mixed breed	Female	12
10	Pug	Female	13
11	Mixed breed	Female	13
12	Golden retriever	Male	14
13	Mixed breed	Male	8
14	Mixed breed	Female	9
15	Golden retriever	Female	8
16	Pitbull	Female	9
17	Terrier mix (unknown)	Male	10
18	Terrier mix (unknown)	Female	10
19	Cocker spaniel	Female	13
20	Golden retriever	Female	8

### Behavioral analysis

#### C-BARQ analysis

In this study, the Mini version of the Canine Behavioral Assessment and Research Questionnaire (C-BARQ) was used to assess behavioral profiles of the dogs. The mini C-BARQ is a shortened, 42-item version of the original C-BARQ that can be completed in under 10 min. This version retains strong agreement with the full C-BARQ and includes 14 behavioral subscales plus nine miscellaneous items (Duffy et al., 2014).

#### Cognitive dysfunction assessment questionnaire

To assess the mental status of the dogs within the scope of this study, DISHAA (Canine Cognitive Dysfunction Rating Scale) developed was used Landsberg and Malamed (2017). This questionnaire focuses on the typical and core symptoms of CCD including disorientation, changes in social interactions, changes in sleep–wake cycles, inappropriate elimination, learning, memory, activity level, and anxiety. Each domain (D, I, S, H, A, A) is rated based on presence/absence of symptoms and frequency of symptoms. According to the DISHAA, a score of 16 or higher would indicate at least moderate decline, and 30 + would indicate severe decline.

### Behavioral assessment for cognitive evaluation

Senior dogs participating in the study were further assessed for cognitive function using the Object Choice Test. The Object Choice Test was conducted in the dogs’ own environments. The entire test procedure was recorded using a video camera (GoPro Hero 6). During the test, especially when the dogs were making a choice between the two objects, caregivers were not permitted to guide or influence their dogs. The experimenter positioned themselves behind the objects, exactly in the middle between the left and right objects. In the Object Choice Test, dogs were required to choose between two different objects to obtain a food reward. Briefly, dogs progressed through stages of increasing difficulty: initial training with visible food placement, followed by two-choice discrimination trials (1–1, 1–2, 2–1, 2–2), where correct choices were rewarded and incorrect choices resulted in no reward. Dogs advancing to the final stage (2–2 discrimination) received an additional point in performance scoring. A detailed protocol including trial structure, reward placement randomization and scoring criteria is provided in Supplementary Methods S1.

#### Helsinki chronic pain index

The Helsinki Chronic Pain Index (HCPI) was used to evaluate chronic pain status in dogs (Hielm-Björkman et al., 2003). This validated instrument assesses mobility, mood changes, vocalizations and behavioral alterations using 11 items. Each item is scored 0–4 based on frequency and severity, yielding total scores from 0 (no pain indicators) to 44 (maximum pain). Scores ≤12 indicate no clinically significant pain, while scores >12 suggest chronic pain (Della Rocca et al., 2023). Caregivers completed the HCPI based on their observations over the preceding week.

#### Quality of life scale

The QOL primarily reflects the observable level of wellbeing in companion animals. For this purpose, dog caregivers were asked a total of 21 questions under 7 main categories to gather information about the quality of life of the dogs participating in the study. These main categories included happiness, mental state, pain, appetite, hygiene, fluid balance and mobility. The scale was scored using a Likert-type scale.

### Hematological analyses

In this study, complete blood count (CBC) and serum-free thyroxine (fT4) analyses were performed to confirm dogs were healthy and did not have underlying endocrine disorders (e.g., hypothyroidism) that could confound behavioral, cognitive, or metabolic assessments. For the hematological analyses, blood samples were collected from the dogs into pediatric tubes containing ethylenediaminetetraacetic acid (EDTA) and into tubes without EDTA (Hematube). The blood collection procedure was conducted in the dogs’ home environment to avoid any additional stress. The collected blood samples were centrifuged at 2,500 rpm for 10 min, and 0.5 mL of serum was transferred to Eppendorf tubes for fT4 analysis. All dogs had fT4 values within normal reference ranges.

#### Serum BDNF analysis

Blood samples collected from the dogs were centrifuged at 2,500 rpm for 10 min. After centrifugation, 0.5 mL of serum was transferred to Eppendorf tubes and stored at −20 °C until the time of analysis. The BDNF analysis was performed using the Cloud Clone 96 T Canine BDNF ELISA kit and conducted using the sandwich ELISA method.

#### Th1/Th2 analysis

Serum samples (0.5 mL) obtained from the blood of the dogs were stored in Eppendorf tubes at −20 °C until flow cytometry analysis. For the flow cytometry analysis, the BioLegend LEGENDplex™ Human Th1/Th2 Panel (8-plex) kit was used. The analysis was performed using a Beckman Coulter CytoFLEX flow cytometer at the Department of Virology, Faculty of Veterinary Medicine, Ankara University.

### Fecal sample collection and analysis

Dog caregivers were provided with sterile fecal collection containers and were instructed to collect their dog’s first morning fecal sample, without letting it touch the ground, using a sterile glove. The collected fecal samples were immediately retrieved from the caregivers and kept on dry ice until they were transported to the laboratory.

#### Fecal microbiota analysis

In this study, fecal microbiota analysis consisted of three main steps: DNA extraction, 16S rRNA gene sequencing using Illumina technology, and real-time PCR. Fecal sample (250 mg) collected from each dog was stored in cryo tubes at −80 °C until DNA extraction. For DNA extraction, the Qiagen DNeasy PowerSoil Pro Kit was used. 16S rRNA Sequencing and Bioinformatic Analysis In 16S rRNA-based metagenomic analyses, a targeted region of the 16S rRNA gene is enriched by PCR, and the resulting PCR products are sequenced. This gene, which is approximately 1,500 base pairs long and encodes ribosomal RNAs, is found in nearly all bacterial and archaeal species. While the gene contains highly conserved regions, it also includes variable regions that are diverse enough to allow phylogenetic classification of different bacterial species. These genetically variable regions are referred to as “variable” regions (e.g., V1, V2, V3, etc.).

Sequence differences in the variable regions of the 16S rRNA gene increase proportionally with evolutionary distance between different species. Therefore, the 16S rRNA sequence is used as a marker for identifying different bacterial species (Hoang et al., 2022).

#### Library preparation

After DNA extraction, the V3–V4 regions of the 16S rRNA gene from each DNA sample were amplified using 16S Forward and 16S Reverse universal eubacterial primers. These primers, used in the comprehensive study by Klindworth et al. (2012), were selected for their high efficiency. In this project, a two-step PCR process was performed during library preparation. For both PCR steps, PrimeSTAR GXL DNA Polymerase (Takara, Japan, #R050B) was used, and for each sample, an individual 25-cycle PCR was performed. The sequencing was performed by Refgen Biotechnology (Turkey) using the NovaSeq 6,000 (Illumina, United States) next-generation sequencing platform and the NovaSeq 6,000 S4 Reagent Kit (Illumina, United States), with paired-end sequencing (2 × 150 bp), following the manufacturer’s guidelines.

#### Bioinformatic analysis

After sequencing, FASTQC was used to perform quality control of the raw sequence reads. Based on the quality control results, the data volume, read quality, GC content distribution, k-mer distribution, and potential adapter contamination of each sample were evaluated. To prevent downstream biases due to low-quality bases and potential adapter/index contamination in the raw reads, a trimming step was performed. For trimming, Trimmomatic (v0.39) was used (Bolger et al., 2014). In this step, low-quality bases at the ends of reads, possible adapter contamination, and chimeric sequences were trimmed based on the Genomes OnLine Database (GOLD) and using Trimmomatic. For taxonomic profiling, the reads were aligned to target organisms using Kraken2 with the RefSeq (Standard-Full, February 2022) database (Wood et al., 2019; Quast et al., 2012). Following alignment, Operational Taxonomic Units (OTUs) were identified for each sample. For calculation of diversity indices, the R:vegan package was used (Oksanen et al., 2019). For data reporting, statistical analyses, and data visualization, R scripts were used.

### Statistical analysis

Statistical analyses were conducted using the SPSS 25.0 software. Descriptive statistics were presented as mean ± standard deviation for continuous variables and frequency (%) for categorical variables. For microbiota analysis, Shannon and Simpson indices were used to calculate diversity. Diversity indices (also known as alpha diversity or phylogenetic metrics) are quantitative measures that reflect both the number of different organisms in a dataset and their phylogenetic relationships (distribution, relatedness, richness). Normalized relative microbial diversity of all samples was visualized using Principal Component Analysis (PCA). Pearson’s correlation was used for normally distributed variables, while Spearman’s rank correlation was applied when normality assumptions were violated. Normality was assessed using the Shapiro–Wilk test (*α* = 0.05) in conjunction with visual inspection of Q-Q plots and histograms. Variables that violated normality assumptions (*p* < 0.05 on Shapiro–Wilk test) or displayed substantial skewness were analyzed using Spearman’s rank correlation. Specifically, Spearman’s correlation was used for the association between *Prevotella copri* relative abundance and HCPI scores due to the right-skewed distribution of *Prevotella copri* abundance across subjects (Shapiro–Wilk: *W* = 0.84, *p* = 0.012) and the ordinal nature of summed HCPI scores. All other correlation analyses employed Pearson’s correlation after normality assumptions were confirmed. A significance level of *p* < 0.05 was considered statistically significant PCA was performed on standardized variables including HCPI scores, DISHAA scores, BDNF levels, Th1/Th2 ratios, and key microbiota phyla to identify dominant multivariate patterns. Components with eigenvalues >1 were retained according to Kaiser’s criterion.

## Results

### Dogs

Out of the 20 dogs initially included in the study, 2 were excluded due to health issues. The average age of the dogs was determined to be 10.68 years (ranging from 8 to 14 years). A total of 12 female and 6 male dogs were included in the study. Unless otherwise specified, all analyses were conducted on *N* = 18 dogs. Fecal microbiota analyses included *N* = 17 dogs (one dog excluded due to inadequate sample collection). The Object Choice Test included *N* = 16 dogs; two dogs were unable to complete the test due to low food motivation (*N* = 1) and mobility limitations (*N* = 1), respectively. All other assessments (C-BARQ, DISHAA, HCPI, QoL, CBC, BDNF, Th1/Th2) included the full cohort of 18 dogs.

### Behavioral evaluation

The C-BARQ analyses were conducted under six main categories: aggression, anxiety, excitability, trainability, separation-related behaviors, and miscellaneous behaviors. According to the results, the average aggression score was 1.33/4.00 (min: 0, max: 4), the average excitability score was 2.11/4.00 (min: 0, max: 4), the average anxiety score was 1.53/4.00 (min: 0, max: 4), the average separation-related behavior score was 0.96/4.00 (min: 0, max: 1), and the average trainability score was 2.22/4.00 (min: 0, max: 4).

### Cognitive function evaluation

*DISHAA:* In questionnaires and behavioral assessments related to cognitive dysfunction, no clinical symptoms of cognitive dysfunction were detected in any of the dogs. According to the DISHAA assessment, scores of 16 or higher indicate moderate cognitive decline, and scores of 30 or higher indicate severe cognitive decline. Based on this evaluation, only one dog showed moderate cognitive decline (DISHAA score: 24, indicating borderline moderate impairment), while the remaining dogs scored below the clinical threshold. The overall average score was 9.05 (min: 2; max: 24).

*Object Choice Test:* In total, 16 dogs participated in the object choice test. While calculating the total score, whether the dogs reached the final stage (2–2) was taken into account. For dogs that reached the final stage, additional 1 point was added to calculate their general performance score (min score = 0; max score = 5). The mean of general performance scores was calculated as 1.94/5 (min = 0, max = 5).

### Pain evaluation

In the HCPI, responses were rated on a scale from 0 to 4. When summed, the total index score ranged from a minimum of 0 to a maximum of 44 (Mölsä et al., 2013). For interpretation of the scale, dogs with an index score between 0 and 12 were considered healthy, while dogs scoring above this threshold were classified as having chronic pain. Based on this evaluation, all of the dogs were found to have chronic pain, with an average pain index of 28.72 (min: 16; max: 43).

### QoL evaluation

This analysis included QoL assessments using a 21-item scale evaluating physical health, behavioral indicators, and emotional wellbeing. Each item was scored on a scale from 0 (very poor/absent) to 4 (very good/normal). The highest mean scores were observed for items indicating positive behaviors and physical wellbeing:

“My dog eats a normal amount of food” (*M* = 3.63, SD = 0.60),

“My dog drinks an adequate amount of water” (*M* = 3.53, SD = 0.61),

“My dog responds to my presence” (*M* = 3.37, SD = 0.83),

“My dog urinates a normal amount” (*M* = 3.42, SD = 0.77).

In contrast, the lowest mean scores were observed for items related to negative states such as pain, depression, or sickness:

“My dog is in pain” (*M* = 1.00, SD = 1.05),

“My dog appears lethargic and/or depressed, not alert” (*M* = 1.05, SD = 1.03),

“My dog breathes quickly and frequently even while resting” (*M* = 0.53, SD = 0.84),

“My dog occasionally trembles and/or shakes” (*M* = 0.37, SD = 0.76).

**Figure 1:**
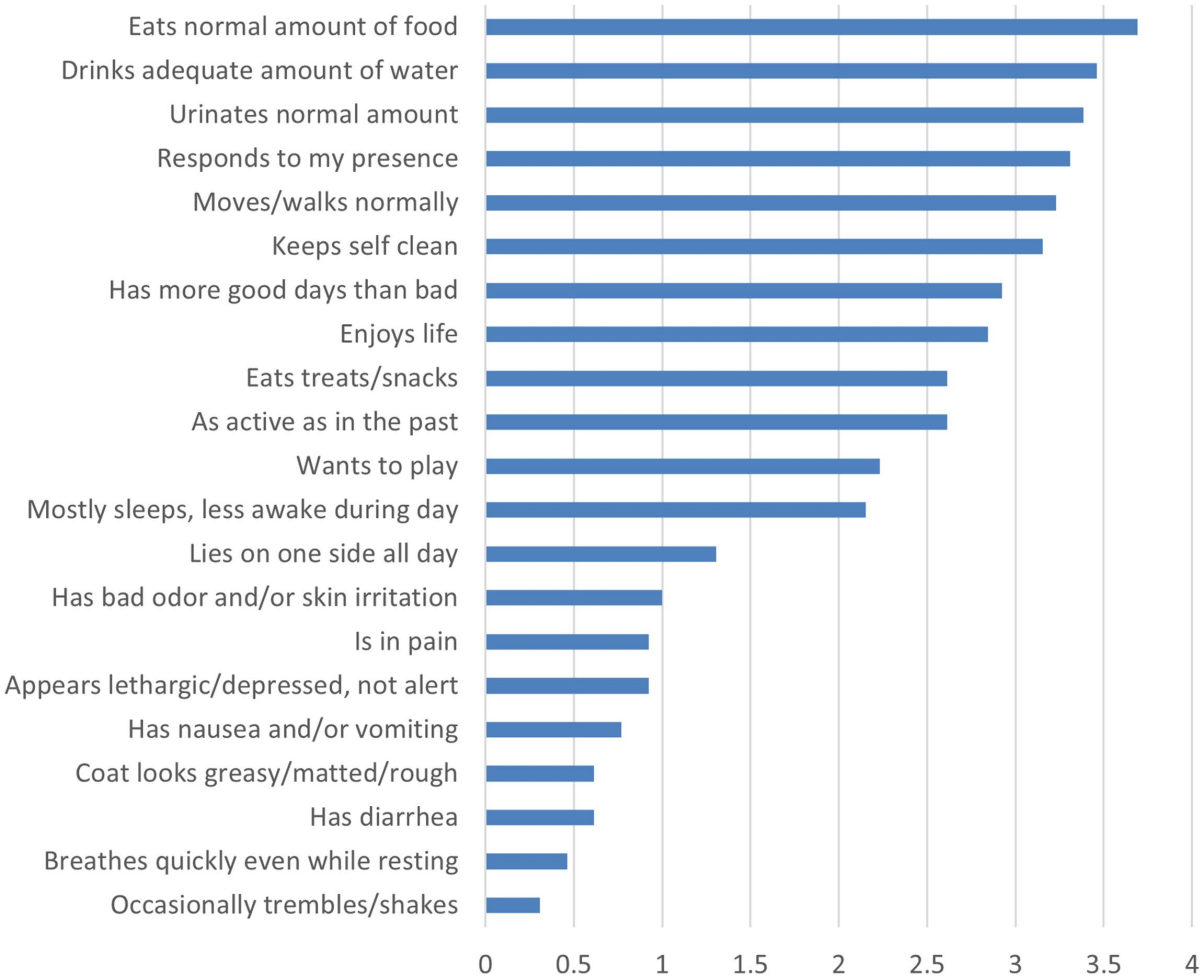
Average mean scores of QoL evaluation.

Mean QoL scores of the dogs for each item are presented in Figure 1.

### Hematological analyses and BDNF levels

**Figure 2:**
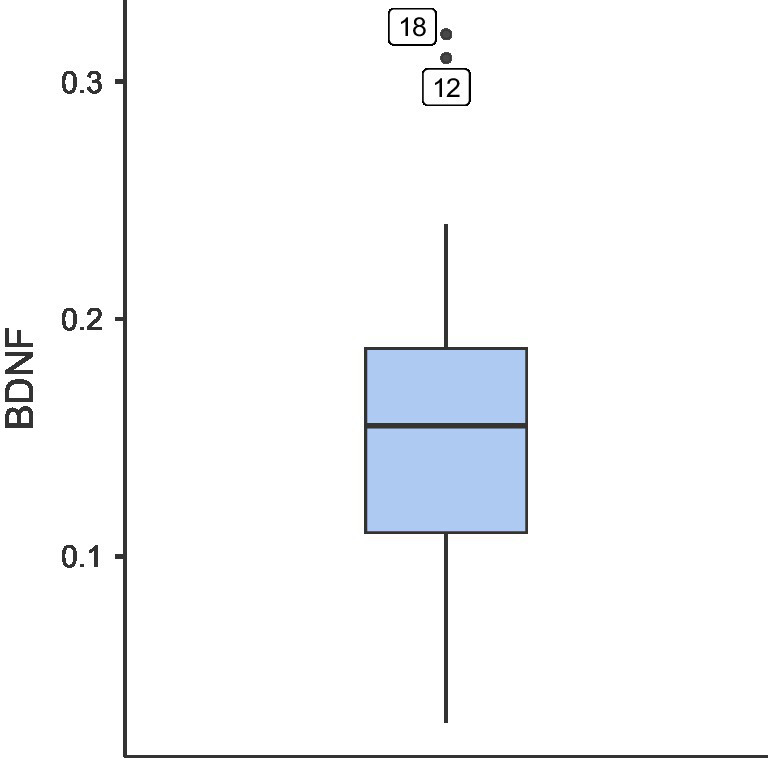
Serum BDNF concentrations (ng/dL) in senior dogs (*N* = 18).

Hemogram analyses revealed normal profiles for most of the dogs (Supplementary file). The average BDNF level in the dogs was determined to be 0.154 ng/dL (SD: 0.082) (Figure 2). In correlation analyses, BDNF levels showed significant associations with several hematological parameters. Specifically, BDNF was positively correlated with red blood cell (RBC) count (*r* = 0.553, *p* = 0.017), while negative correlations were observed with mean corpuscular hemoglobin concentration (MCHC) (*r* = −0.512, *p* = 0.030), mean corpuscular hemoglobin (MCH) (*r* = −0.553, *p* = 0.017), and mean corpuscular volume (MCV) (*r* = −0.471, *p* = 0.049).

### Th1/Th2 levels

**Figure 3:**
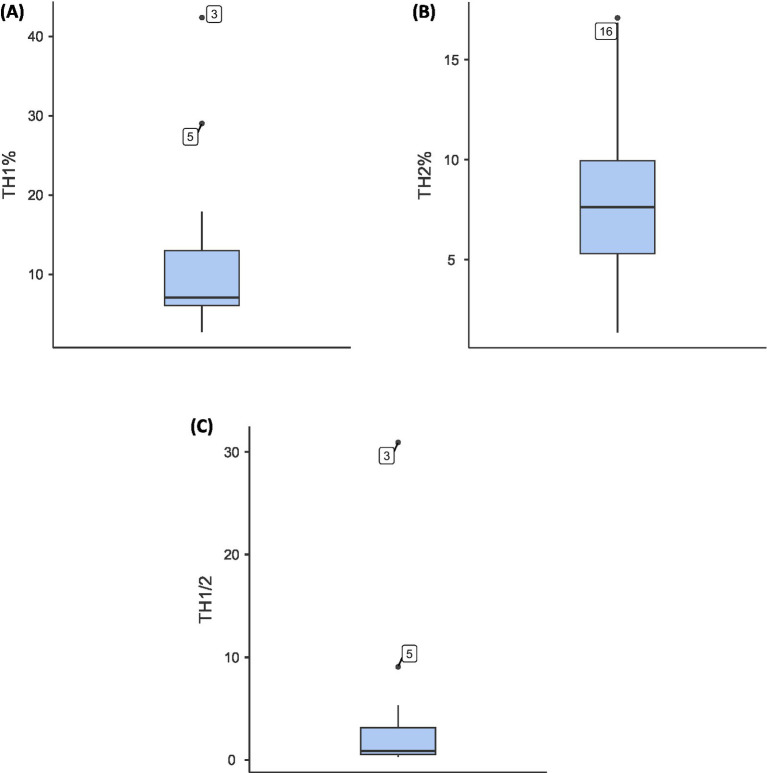
**(A–C)** Boxplots showing distribution of **(A)** Th1 percentages, **(B)** Th2 percentages, and **(C)** Th1/Th2 ratios in senior dogs with chronic pain (*N* = 18).

Th1/Th2 immune profiling revealed substantial inter-individual variability (Figure 3). Th1% ranged from 2.75 to 42.4% (mean ± SD: 11.3 ± 9.95%), while Th2% ranged from 1.37 to 17.09% (mean: 7.90 ± 4.44%). The Th1/Th2 ratio showed a right-skewed distribution (median: 1.52; IQR: 0.85–3.21; range: 0.32–30.9) with one extreme outlier (30.9) that substantially inflated the mean (3.57 ± 7.23). Given this skewness, the median may better represent the typical Th1/Th2 balance in this cohort. Box plot and distribution visualizations are presented in Figure 3.

### Gut microbiota analysis

#### 16S rRNA analysis results

**Figure 4:**
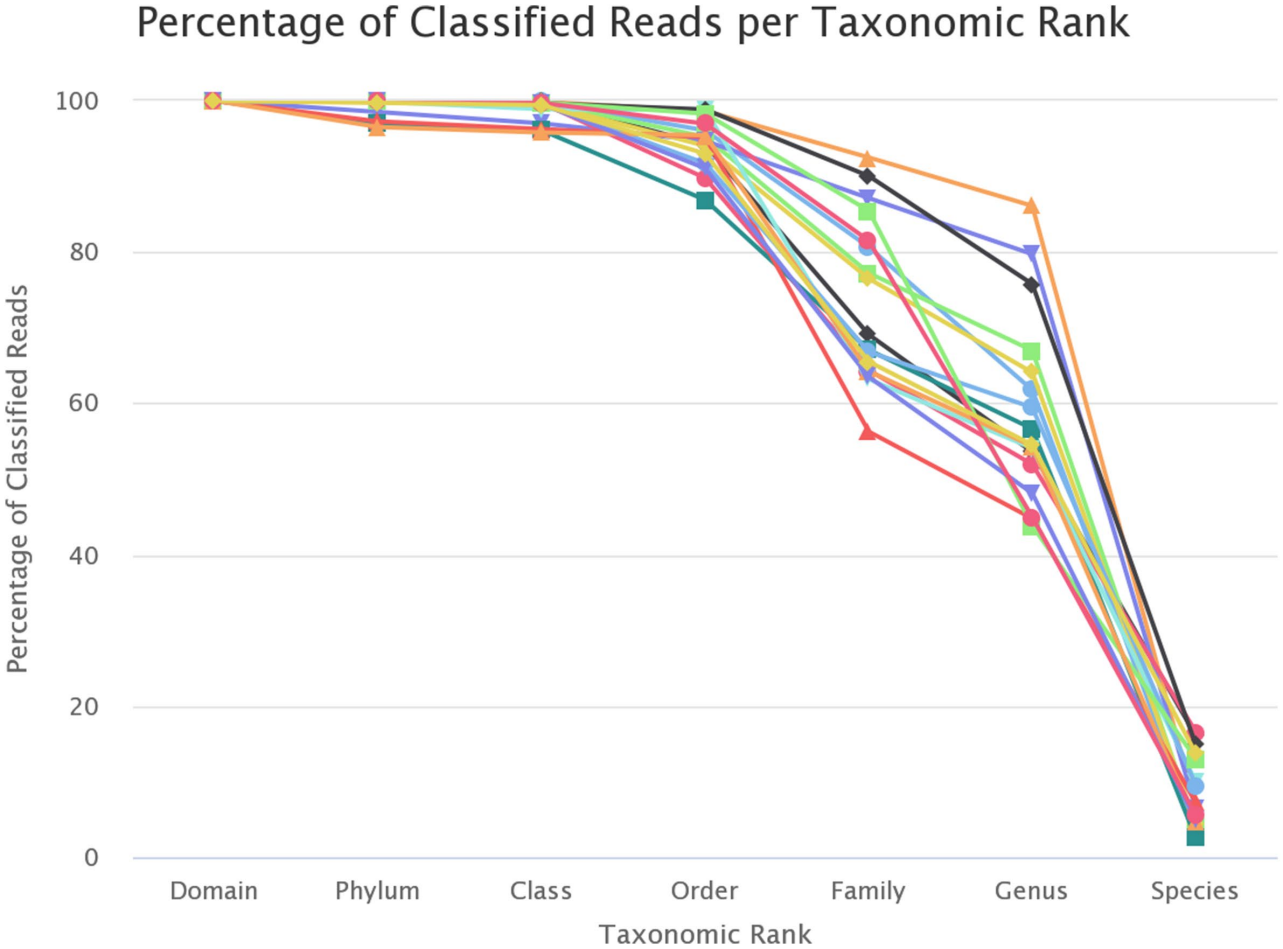
Identified taxonomic levels.

The reads that passed quality control were assigned to the taxonomic unit with the highest similarity at various taxonomic levels. Figure 4 shows the proportion of reads from each sample that could be assigned to units at different taxonomic levels.

**Figure 5:**
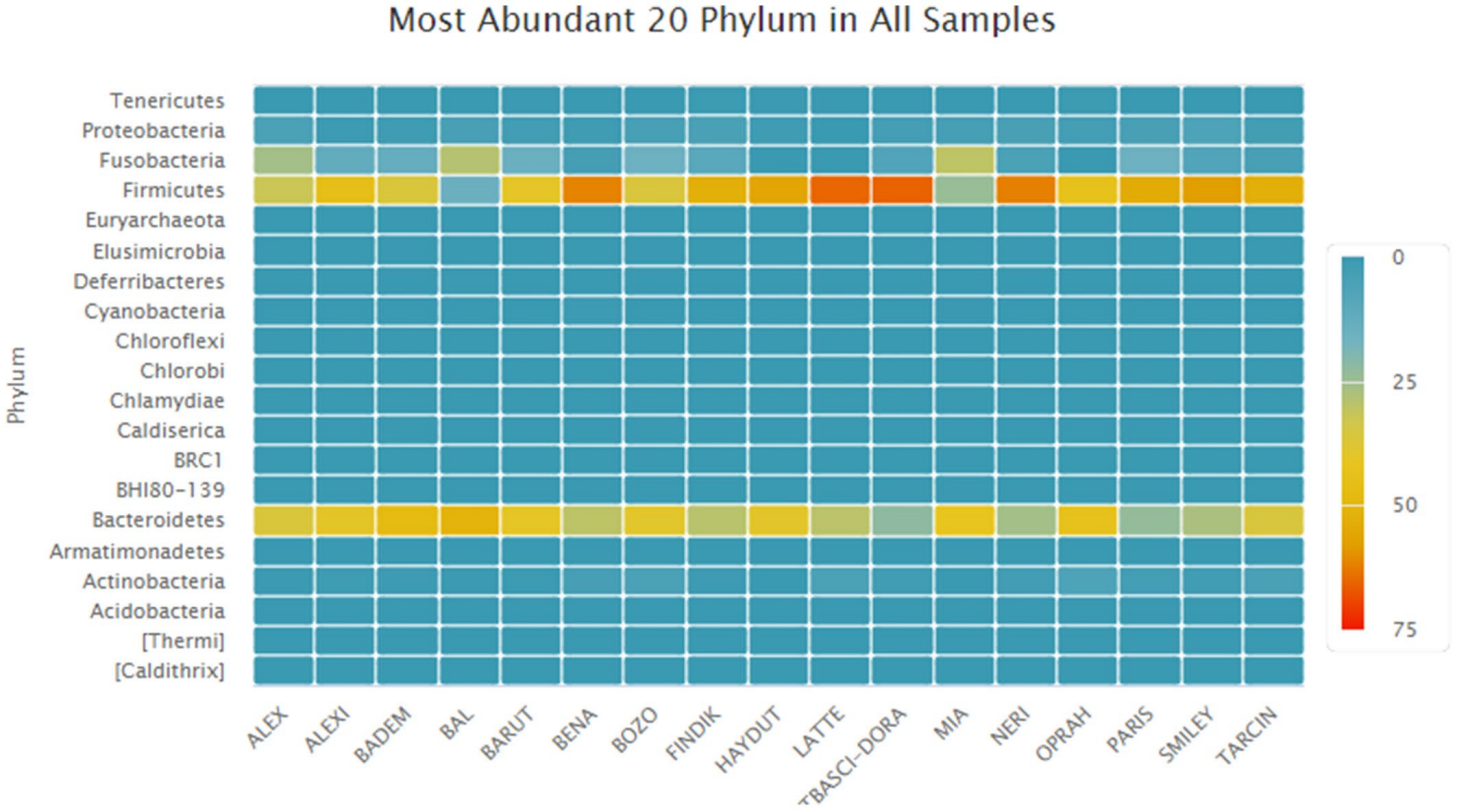
Distributions of the most abundant phyla.

*Phylum Level:* Metagenomic analysis revealed a total of 50 phyla across all samples. The top 20 most abundant phyla are presented in a heatmap in Figure 5. The five main phyla observed across the entire sample group were Firmicutes (47.16% ± 14.34), Bacteroidetes (36.6% ± 8.35), Fusobacteria (11.26% ± 9.45), Proteobacteria (2.99% ± 1.66), and Actinobacteria (2.14% ± 1.87).

*Class level:* A total of 128 classes were identified across all samples. The most abundant class was Clostridia (40.63% ± 14.7). The top 5 classes found were Clostridia, Bacteroidia, Fusobacteriia, Bacilli, and Erysipelotrichi.

*Order level:* A total of 206 orders were identified across all samples. The five most abundant orders in the samples were Bacteroidales, Clostridiales, Fusobacteriales, Erysipelotrichales, and Coriobacteriales. The most abundant order was Bacteroidales (36.38% ± 8.61).

*Family level:* A total of 288 families were identified across all samples. The five most abundant families in the samples were Bacteroidaceae, Fusobacteriaceae, Veillonellaceae, Prevotellaceae, and Lachnospiraceae. The most abundant family was Bacteroidaceae (52.45% ± 12.08).

*Genus level:* A total of 488 genera were identified across all samples. The five most abundant genera in the samples were Bacteroides, Fusobacterium, Prevotella, Turicibacter, and Collinsella. The most abundant genus was Bacteroides (23.03% ± 12.08).

**Figure 6:**
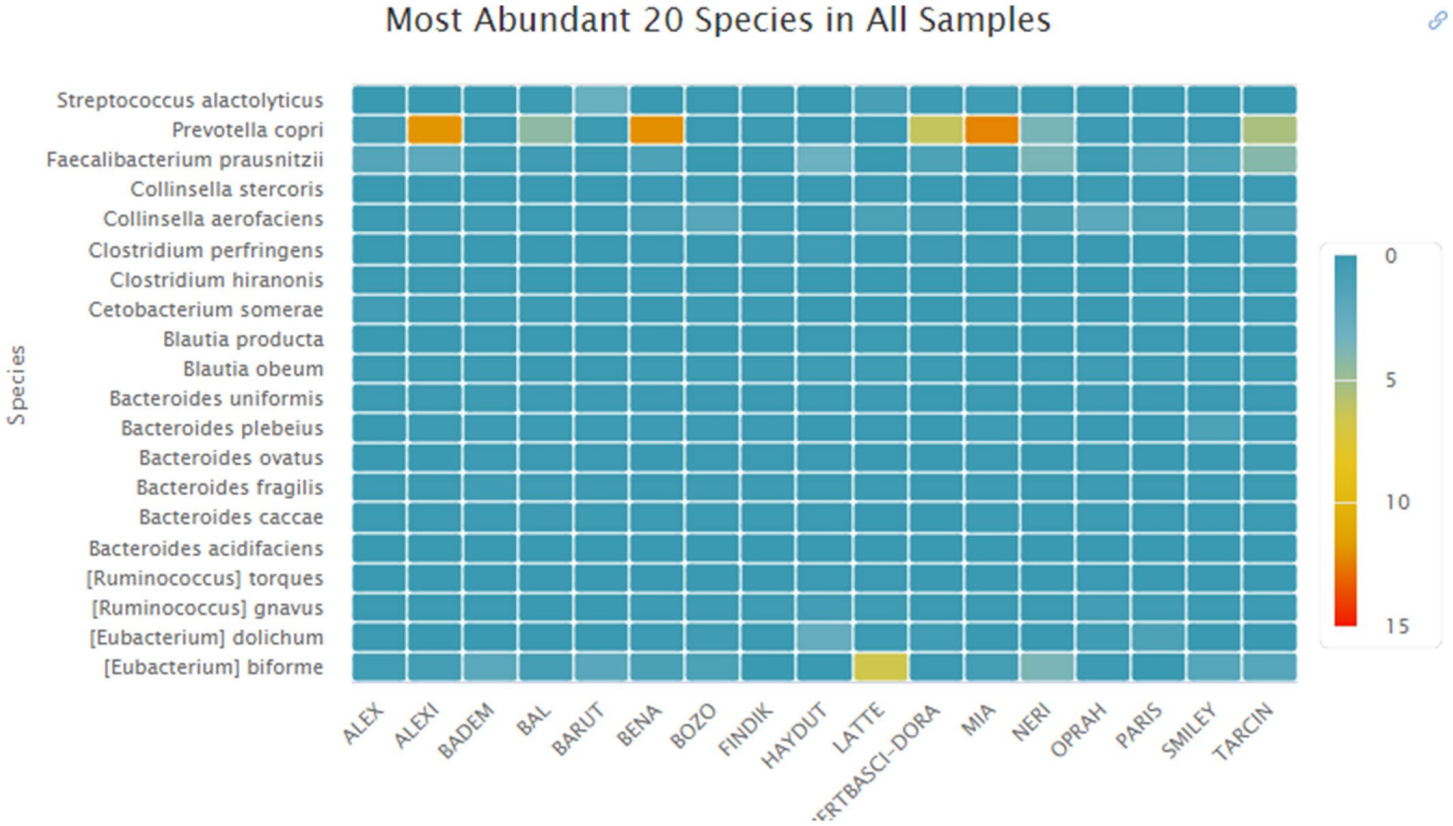
Distributions of the most abundant species.

*Species level:* A total of 215 species were identified across all samples. The five most abundant species in the samples were *Prevotella copri*, *Eubacterium biforme*, *Faecalibacterium prausnitzii*, *Collinsella aerofaciens*, and *Eubacterium dolichum*. The most abundant species was *Prevotella copri* (3.36%) (Figure 6).

Correlation analysis was conducted to examine the relationship between the relative abundance of *Prevotella copri* and pain scores assessed by the Helsinki Chronic Pain Index (HCPI) in senior dogs. Spearman’s rank correlation indicated a moderate, non-significant positive association between pain scores and *Prevotella copri* abundance (*ρ* = 0.47, *p* = 0.064).

### Multivariate integration of pain, neurotrophic, immune, and microbiota variables

Variables included in the PCA were selected based on three criteria: (1) theoretical relevance to the hypothesized pain-immune-microbiota axis based on prior literature (Honda and Littman, 2016; Goudman et al., 2024); (2) complete or near-complete data availability across subjects to maintain analytical power; and (3) representation of distinct biological domains (behavioral: DISHAA, HCPI; neurotrophic: BDNF; immunological: Th1%, Th2%, Th1/Th2 ratio; microbiota: dominant phyla including Firmicutes, Bacteroidetes, Proteobacteria, and key taxa of interest including Prevotella). This multi-domain approach was designed to capture the multifactorial nature of aging while maintaining interpretability given our exploratory sample size.

The scree plot (eigenvalue analysis) of PCA indicated that the first two principal components together accounted for 52.4% of total variance (PC1: 30.2%; PC2: 22.2%), meeting Kaiser’s criterion (eigenvalues >1). The remaining 47.6% of variance was distributed across eight additional components with eigenvalues <1, indicating substantial biological heterogeneity not captured by the measured variables. Components 3 (15.8% variance) and 4 (13.1% variance) also exceeded Kaiser’s criterion (eigenvalues >1), collectively explaining an additional 28.9% of variance. PC3 was primarily driven by BDNF levels and specific hematological parameters, which reflects a neurotrophic-hematological axis independent of the pain-immune patterns captured in PC1. PC4 showed complex loadings across multiple microbiota taxa without clear biological interpretation. The presence of these additional components with eigenvalues >1 indicates substantial biological heterogeneity in the cohort that extends beyond the primary pain-microbiota-immune relationships. However, given the exploratory nature of this study and our small sample size, we focused interpretation on the first two components, which together explained the majority (52.4%) of captured variance and demonstrated the most interpretable biological patterns. Future studies with larger samples are needed to validate and further characterize these secondary dimensions of variation.

### Principal component 1 (30.2% variance): pain-microbiota-immune axis

**Figure 7:**
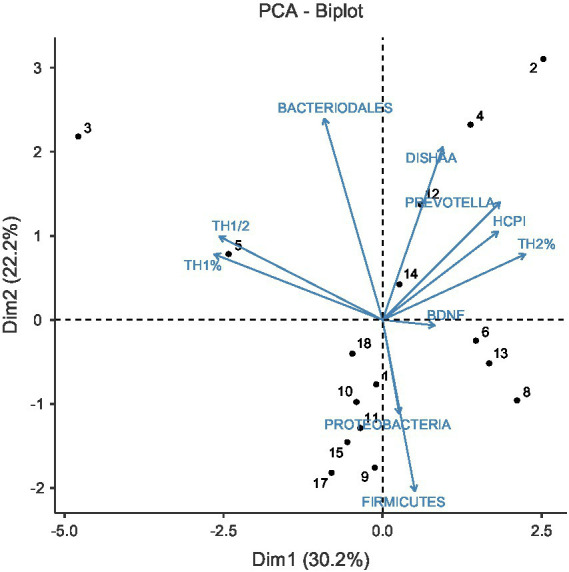
PCA illustrating multivariate relationships among pain, neurotrophic, immune, and microbiota variables in senior dogs.

Examination of the PCA biplot (Variables-PCA and PCA-Biplot) reveals that PC1 (Dim 1 on x-axis) is characterized by opposing patterns along a horizontal axis (Figure 7). Variables loading positively (toward the right) on PC1 include HCPI, Prevotella, Th2%, and DISHAA, while variables loading negatively (toward the left) include Th1/Th2 ratio and Th1%. The microbiota variables show more complex positioning: Firmicutes and Proteobacteria load negatively, while Bacteroidales loads positively.

Dogs positioned at the positive extreme of PC1 (e.g., dogs 4, 6, 13 based on the Individuals-PCA plot) exhibited higher scores on pain (HCPI), cognitive dysfunction (DISHAA), Prevotella abundance, and Th2%, with lower Th1/Th2 ratios. Conversely, dogs at the negative extreme (e.g., dog 3) showed the opposite pattern. BDNF is positioned very close to the origin in the biplot, indicating minimal contribution to PC1.

### Principal component 2 (22.2% variance): the microbiota composition axis

PC2 reveals a distinct dimension of variation primarily characterized by gut microbiota composition. Bacteroidales loads strongly positive on PC2, while Firmicutes and Proteobacteria load strongly negative. This represents an axis of microbiota composition independent of the pain-immune-cognitive patterns captured by PC1.

Dogs positioned at the positive extreme of PC2 (e.g., dogs 3, 4) exhibit Bacteroidales-dominated microbiota profiles, while those at the negative extreme (e.g., dogs 8, 9, 15, 17) show Firmicutes/Proteobacteria-dominated profiles.

## Discussion

This exploratory study integrated behavioral, cognitive, immunological, neurotrophic, and microbiota assessments to characterize aging-related changes in senior companion dogs. Our findings suggest complex multivariate relationships among chronic pain, immune profiles, neurotrophic signaling and gut microbiota composition in senior dogs.

An interesting finding of our study was the discrepancy between objective pain indices and caregiver perceptions. Despite all dogs scoring above the chronic pain threshold, caregiver-reported quality of life ratings were generally high, with particularly low scores on the direct pain assessment item (“My dog is in pain”: *M* = 1.00, SD = 1.05 on a 0–4 scale). This disconnect between objective and subjective pain assessments aligns with previous evidence that chronic pain in dogs is frequently under-recognized and may be misattributed to normal aging (Belshaw and Yeates, 2018; Demirtas et al., 2023). Several factors may contribute to this discrepancy. HCPI primarily assess observable mobility changes, which caregivers may normalize as age-appropriate (Della Rocca et al., 2023; Hielm-Björkman et al., 2003), while QoL instruments may fail to capture chronic discomfort that develops gradually (Mich and Hellyer, 2008). Additionally, dogs’ natural tendency to mask discomfort may complicate recognition of subtle pain related behavioral changes (Mills et al., 2020). This shows the essential value of integrating objective physiological biomarkers into routine geriatric screening (Belshaw and Yeates, 2018; Pais et al., 2024).

The mean BDNF level in our study sample was markedly lower than values typically reported in dogs. While Isaka et al. (2021) reported no significant age-related BDNF decline in dogs, this discrepancy may reflect a fundamental difference in pain status between samples. In humans, chronic musculoskeletal pain have been found to be associated with reduced plasma BDNF concentrations, with lower levels correlating with greater pain severity (Di-Bonaventura et al., 2025; Xiong et al., 2024; Ortolá et al., 2025). These reductions reflect impaired neuroplasticity, altered pain modulation, and chronic inflammation (Dimmek et al., 2021). Given BDNF’s critical role in synaptic plasticity and central sensitization pathways (Huang and Reichardt, 2001), one may suggest that reduced BDNF availability may both result from and contribute to maladaptive pain processing.

The significant correlations between BDNF and hematological parameters (positive with RBC; negative with MCV, MCH, MCHC) are consistent with human studies and supported by our multivariate PCA findings. Specifically, PC3 was primarily driven by BDNF levels and hematological parameters, which reflects a neurotrophic-hematological axis independent of pain-immune patterns (PC1). These correlations suggest a state of chronic, low-grade inflammation that modulates both BDNF levels and erythrocyte morphology through oxidative stress and pro-inflammatory cytokine signaling (McSorley et al., 2019; Cheng et al., 2020; Buranakarl et al., 2009; Tsai, 2015). The emergence of PC3 as a distinct component further suggests that neurotrophic-hematological interactions represent a parallel pathway of age-related pathology. While the absence of pain-free senior or young control cohorts precludes the definitive separation of chronological from pathological aging, the universal chronic pain status of this sample, aligned with established BDNF-pain paradigms in humans, suggests that reduced BDNF levels are more likely driven by pain-related inflammatory mechanisms than by physiological aging alone (Gladyshev and Gladyshev, 2016).

Contrary to aging laboratory Beagles showing increased Th1 percentages with age (Horiuchi et al., 2007), our dogs exhibited lower Th1/Th2 ratios with reduced Th1 and elevated Th2 percentages. This divergence may reflect differences between laboratory-bred Beagles under controlled conditions versus diverse client-owned breeds experiencing varied environmental stressors, diets and antigenic exposures (Hekman et al., 2014; Mârza et al., 2024). However, given the high prevalence of chronic pain, this pattern likely reflects pain-associated immune dysregulation. Increased Th2 responses are recognized modulators of chronic pain (Yu and Wang, 2022), contrasting with typical immunosenescence characterized by Th1 skewing and inflammaging (Sakata-Kaneko et al., 2000; Martynova et al., 2022; Xia et al., 2016; Goronzy and Weyand, 2017; Liu et al., 2023). The PCA analysis revealed that dogs at the positive extreme of PC1 exhibited higher pain scores, elevated DISHAA scores and Th2-skewed immunity. This pattern suggests a more pathological aging profile, while those at the negative extreme showed relatively lower pain and cognitive dysfunction scores with more Th1-dominant profiles.

Metagenomic analysis in our study revealed Firmicutes and Bacteroidetes as dominant phyla, consistent with previous studies (Fernández-Pinteño et al., 2023; Pilla and Suchodolski, 2021). The relative decline in Fusobacteria aligns with aging dogs (Mizukami et al., 2019) but diverges from human aging patterns (Biagi et al., 2010). This result shows species-specific differences despite dogs’ greater microbial similarity to humans than to laboratory rodents (Coelho et al., 2018; Misic et al., 2015; Deng and Swanson, 2014).

Recent research has linked gut microbiota composition, particularly Firmicutes abundance, to behavioral phenotypes in dogs, especially in relation to anxiety and aggression (Kirchoff et al., 2019; Craddock et al., 2022; Sacchettino et al., 2025). In our PCA, Firmicutes loaded negatively on both PC1 and PC2, opposing the pain–Th2–Prevotella cluster. Although overt behavioral pathology was not a defining feature of our sample, it is important to note that the universal presence of chronic pain may shape gut microbial communities through altered autonomic tone, stress hormone signaling, and inflammation (Camps et al., 2012; Mills et al., 2020; Malkani et al., 2024; Guo et al., 2019; Goudman et al., 2024).

The observed abundance of Lachnospiraceae (3.75%) was notably lower than expected, as this family is consistently reported among the most abundant taxa in healthy dogs (Pilla and Suchodolski, 2021). As primary producers of short-chain fatty acids, particularly butyrate, Lachnospiraceae exert anti-inflammatory, neuroprotective, and gut barrier-protective effects (Honda and Littman, 2016). In humans, reduced Lachnospiraceae abundance links to cognitive decline in Alzheimer’s disease through loss of butyrate-mediated neuroprotection and impaired blood–brain barrier integrity (Zhuang et al., 2018; Li et al., 2022). The low levels in our senior dogs may, therefore, indicate early subclinical alterations in gut-brain axis signaling. However, alternative explanations such as pain-associated dysbiosis, dietary influences or breed-specific patterns cannot be excluded (Wang et al., 2019; Pilla and Suchodolski, 2021).

A trend toward a positive association between *Prevotella copri* abundance and chronic pain scores was observed and warrants attention. *Prevotella copri* has been implicated in inflammatory conditions, particularly rheumatoid arthritis, where its expansion correlates with disease activity and autoantibody production (Scher et al., 2013; Maeda et al., 2016; Maeda and Takeda, 2017; Pianta et al., 2017). Consistent with this literature, the multivariate co-loading of *Prevotella copri*, Th2 polarization, and pain severity in our PCA suggests a biologically meaningful association, although causal relationships cannot be inferred from the present data.

Similarly, the low abundance of *Clostridium hiranonis*, essential for secondary bile acid metabolism and depleted in inflammatory enteropathies (AlShawaqfeh et al., 2017; Pilla et al., 2020), may indicate early shifts in gastrointestinal homeostasis.

A Pain-Microbiota-Immune Axis (PC1) in our PCA analyses suggests mechanistic interconnections mediated through the gut-brain-immune axis. Chronic pain can drive gut dysbiosis via altered autonomic tone and HPA-axis-mediated changes in gut permeability, which may sustain a “leaky gut” phenotype where bacterial metabolite translocation promotes systemic inflammation, further exacerbating neuroinflammation, pain sensitivity, and cognitive decline (Goudman et al., 2024; Guo et al., 2019; Ragonnaud and Biragyn, 2021; Gupta et al., 2024). This suggests that interventions targeting gut dysbiosis (dietary modulation, prebiotics, probiotics, synbiotics) and BDNF-enhancing strategies (exercise, environmental enrichment, pharmacological interventions) may offer novel therapeutic approaches for managing chronic pain and inflammation in senior dogs (Goudman et al., 2024; Ragonnaud and Biragyn, 2021; Lu et al., 2013; Gliwińska et al., 2023).

PC2 revealed microbiota compositional variation (Bacteroidetes vs. Firmicutes/Proteobacteria balance) that operated independently of pain-immune-cognitive patterns. This pattern suggests an influence from factors not fully captured in our assessment such as diet, medication history, environmental exposures and breed-specific factors (Coelho et al., 2018; Wang et al., 2019; Redondo-Useros et al., 2020; Fernández-Pinteño et al., 2023; Sacchettino et al., 2025). Overall, the PCA suggests that aging-related biological changes exist along continuous gradients rather than discrete healthy versus pathological categories.

The discordance between caregiver-completed DISHAA questionnaires and objective Object Choice Test performance suggests that objective cognitive tests may be more sensitive to early changes than caregiver observation-based questionnaires. Objective tests directly assess executive function, working memory, and discrimination learning in controlled environments, whereas DISHAA relies on caregiver detection of behavioral changes in everyday contexts (Landsberg and Malamed, 2017; Wallis et al., 2014; Kubinyi and Iotchev, 2020; Mongillo et al., 2013). However, given the universal chronic pain status of our sample, low Object Choice Test scores may reflect pain-related deficits in motivation, attention, or physical comfort rather than true cognitive impairment (Mills et al., 2020; Malkani et al., 2024; Camps et al., 2012). The minimal contribution of DISHAA and BDNF to the primary principal components supports the interpretation that pain, rather than cognitive decline, may be the dominant factor influencing objective test performance.

### Limitations

Several limitations must be acknowledged when interpreting these findings. First, the small sample size limits statistical power to detect subtle associations and restricts generalizability. These preliminary results should be interpreted with caution and validated in larger cohorts. Second, the inclusion of diverse breeds introduces genetic, morphological and physiological heterogeneity that may confound interpretation of aging-related outcomes. While this heterogeneity reflects the real-world diversity of companion dog populations and enhances external validity, it complicates attribution of findings to aging processes independent of breed-specific factors. Future studies designed to focus on individual breeds or to incorporate breed-stratified approaches will be necessary to disentangle aging-related effects from breed-associated variation. Third, and most critically, the absence of a control group of pain-free senior dogs fundamentally limits causal inference. While our multivariate patterns suggest that pain plays a significant role, these findings must be considered preliminary and hypothesis-generating. Future studies incorporating three comparison groups (young healthy dogs, senior pain-free dogs and senior dogs with chronic pain) are essential to further investigate these relationships. Fourth, diet was not systematically documented in this study, although the profound influence of dietary composition on gut microbiota, immune function, and metabolic health is well-established (Wang et al., 2019; Coelho et al., 2018). All dogs were fed commercial diets, but brands, formulations, and feeding regimens varied across individuals. In addition, feeding bowl hygiene represents a potential environmental confounder, as variations in cleaning practices, bowl materials and feeding habits have been shown to influence microbial contamination and may affect gut microbiota composition (Raspa et al., 2023); however, this factor was not systematically assessed in the present study. Finally, our PCA was conducted on a sample size below conventional recommendations for the number of variables included, which limits the stability and generalizability of the identified components. Although the retained components met Kaiser’s criterion (eigenvalues >1), a standard threshold indicating meaningful variance extraction, small sample sizes increase the risk of unstable component structures and overfitting. These multivariate patterns should be interpreted as preliminary and require validation in substantially larger cohorts to confirm the reproducibility of the observed pain-immune-microbiota relationships.

Despite these limitations, this study provides valuable preliminary insights into the complex interplay among pain, cognition, immune function, and gut microbiota in senior companion dogs. The integrated, multivariate approach adopted here represents a methodological advance in canine aging research and offers a framework for identifying candidate biomarkers of pathological versus healthy aging.

## Conclusion

This exploratory study suggests that aging in dogs is characterized by coordinated, system-level changes across the gut microbiota, immune balance, and neurotrophic signaling, with chronic pain emerging as a potentially central organizing feature of biological variation in our cohort. Alterations in specific microbial taxa, circulating BDNF levels and Th2-skewed immune profiles together formed a constellation of biological signatures that co-varied with chronic pain indices, despite the absence of diagnosed systemic disease and in the context of caregiver reports indicating generally good quality of life.

The continuous distribution of individual dogs across multivariate PCA dimensions further suggests that aging-related biological changes do not segregate into discrete healthy versus pathological categories, but instead unfold along multidimensional gradients. These findings support future comparative and longitudinal studies incorporating young dogs, pain-free senior dogs, and seniors with chronic pain to validate candidate biomarkers, clarify causal pathways, and develop targeted strategies for earlier welfare screening and intervention in aging companion dogs.

## Data Availability

The original contributions presented in the study are publicly available. This data can be found here: NCBI BioProject accession number PRJNA1422677 (http://www.ncbi.nlm.nih.gov/bioproject/1422677), with associated BioSample accessions SAMN55315947-SAMN55315956.
