# Supplementary material for: An exploratory study of behavioral, cognitive, physiological, and microbiota profiles in senior dogs
Source: Front Behav Neurosci. 2026 Feb 24;20:1689807. doi: 10.3389/fnbeh.2026.1689807 (PMC12990210; doi:10.3389/fnbeh.2026.1689807)
Supplement: Supplementary file 1 [file Table_1.docx]

**Object Choice Test**

The testing procedure was as follows:

1. Two identical, opaque objects (bowls) of the same color, size, and shape were placed upside-down on a flat surface.
2. A food reward was placed under one of the objects. It was important that the dog could see which side the food was placed on.
3. Initially, the reward was always placed under the same object (e.g. always under the right-side bowl). The dog was then encouraged to approach the object and either touch it with its nose or paw. Each trial allowed up to two choices. If the dog chose the incorrect (empty) bowl, the trial was ended. This phase was repeated for five trials.
4. In the second phase of the test, the reward was removed. The dog was expected to approach the same side (e.g. the right bowl) where the food had previously been placed, even though no food was present. This phase was also repeated for five trials.

To test memory retention after a delay, the entire procedure was repeated after a 15-minute interval. In the performance assessment scoring, the criterion was based on the dogs demonstrating performance at all test stages and progressing to the final stage.
 The entire test procedure was divided into four stages: 1-1, 1-2, 2-1, and 2-2.

***Stage 1-1*** *refers to the initial phase of the object choice test, where a reward treat was placed (1 pt).****Stage 1-2*** *followed stage 1-1 and was conducted without the reward treat (1 pt).*

***Stage 2-1*** *was conducted 15 minutes after the completion of the first stages and again included a reward treat (1 pt).****Stage 2-2*** *was the final stage of the object choice test, conducted immediately after stage 2-1, and without the reward treat (1 pt).*

Each dog that successfully performed at a given stage was awarded 1 point for that stage. Dogs that successfully performed in the final stage (2-2) were given an additional +1 point to calculate general performance score.

**Hemogram results**
